# Gamma oscillation in functional brain networks is involved in the spontaneous remission of depressive behavior induced by chronic restraint stress in mice

**DOI:** 10.1186/s12868-016-0239-x

**Published:** 2016-01-12

**Authors:** Arshi Khalid, Byung Sun Kim, Bo Am Seo, Soon-Tae Lee, Keun-Hwa Jung, Kon Chu, Sang Kun Lee, Daejong Jeon

**Affiliations:** Department of Bio and Brain Engineering, Korea Advanced Institute of Science and Technology, 291 Daehak-ro, Yuseong, Daejeon, 305-701 Republic of Korea; Laboratory for Neurotherapeutics, Department of Neurology, Comprehensive Epilepsy Center, Biomedical Research Institute, Seoul National University Hospital (SNUH), 101 Daehak-ro, Jongno-gu, Seoul, 110-744 Republic of Korea; Advanced Neural Technologies, Ihwhajang-gil 71, Jongno-gu, Seoul, 03087 Republic of Korea

**Keywords:** Chronic restraint stress, Depression, Behavioral remission, Mouse, Electroencephalography, Functional connectivity, Persistent brain network homology, Gamma oscillation

## Abstract

**Background:**

Depression is one of the most prevalent mood disorders, and is known to be associated with abnormal functional connectivity in neural networks of the brain. Interestingly, a significant proportion of patients with depression experience spontaneous remission without any treatment. However, the relationship between electroencephalographic (EEG) functional connectivity and the spontaneous remission in depression remains poorly understood. Here, we investigated regional and network brain activity using EEG signals from a chronic restraint stress (CRS)-induced mouse model of depression. After 1 (CRS1W) or 3 weeks (CRS3W) following the cessation of a 4-week-long CRS, mice were subjected to depression-associated behavioral tasks. EEG signals were obtained from eight cortical regions (frontal, somatosensory, parietal, and visual cortices in each hemisphere).

**Results:**

The CRS1W group exhibited behavioral dysfunctions in the open field and forced swim tasks, whereas the CRS3W group displayed normal levels of behaviors in those tasks. In a linear correlation analysis, the CRS1W group exhibited increased correlation coefficient values at all frequency bands (delta, 1.5–4; theta, 4–8; alpha, 8–12; beta, 12–30; gamma, 30–80 Hz) compared with the control group. However, the differences in delta- and gamma-frequency bands between the control and CRS1W groups were no longer observed in the CRS3W group. Persistent brain network homology revealed significantly different functional connectivity between the control and CRS1W groups, and it demonstrated a huge restoration of the decreased distances in the gamma-frequency band for the CRS3W group. Moreover, the CRS3W group displayed a similar strength of connectivity among somatosensory and frontal cortices as the control group.

**Conclusion:**

A mouse model of CRS-induced depression showed spontaneous behavioral remission of depressive behavior. Using persistent brain network homology analysis of EEG signals from eight cortical regions, we found that restoration of gamma activity at the network level is associated with behavioral remission.

**Electronic supplementary material:**

The online version of this article (doi:10.1186/s12868-016-0239-x) contains supplementary material, which is available to authorized users.

## Background

Depression is a common mental disorder that affects approximately 121 million people worldwide, and is considered one of the leading causes of disability. This disorder is associated with increased physical illness, decreased social functioning, and a high mortality rate, resulting in huge social and economic strain [1]. Interestingly, there is a relatively large group of individuals who show remission from depression without treatment [2, 3]. The rate of this spontaneous remission was high among depressed individuals who were on a waitlist for participation in a clinical study, or who served as a control in a clinical study: 23 % of adults were in remission within 3 months without formal treatment, 32 % were in remission within 6 months, and 53 % were in remission within 1 year [3]. Although the proportions remitted are known to decrease as depression severity increases [4, 5], spontaneous remission from untreated depression remains an overlooked area of research.

Many studies have reported that numerous regions of the brain are affected by depression, and that the symptoms of depression are associated with the dysregulation of distributed neural networks, encompassing cortical regions, rather than the functional breakdown of a single discrete brain region [6, 7]. Electroencephalographic (EEG) signals have been used in the analysis of functional connectivity in humans and animals [8–10]. The recovery from aberrant EEG functional connectivity in neural networks in the brain is used as a biomarker of treatment outcome in depression [11–15]. However, there have been no EEG studies concerning functional connectivity at the network level in the brain during spontaneous remission from depression.

In this study, we investigated functional connectivity by applying persistent brain network homology, a multi-scale network-modeling framework, to EEG data from eight cortical regions (frontal, somatosensory, parietal, and visual cortices in each hemisphere) of a chronic restraint stress (CRS)-induced mouse model of depression [9, 16]. The physical stress caused by chronic restraint is known to induce depression-like behaviors and neurochemical changes in rodents, and it has been shown that chronic treatment with antidepressants, such as fluoxetine, can reverse the depression-like phenotype of the CRS model [17–19].

## Results

**Fig. 1:**
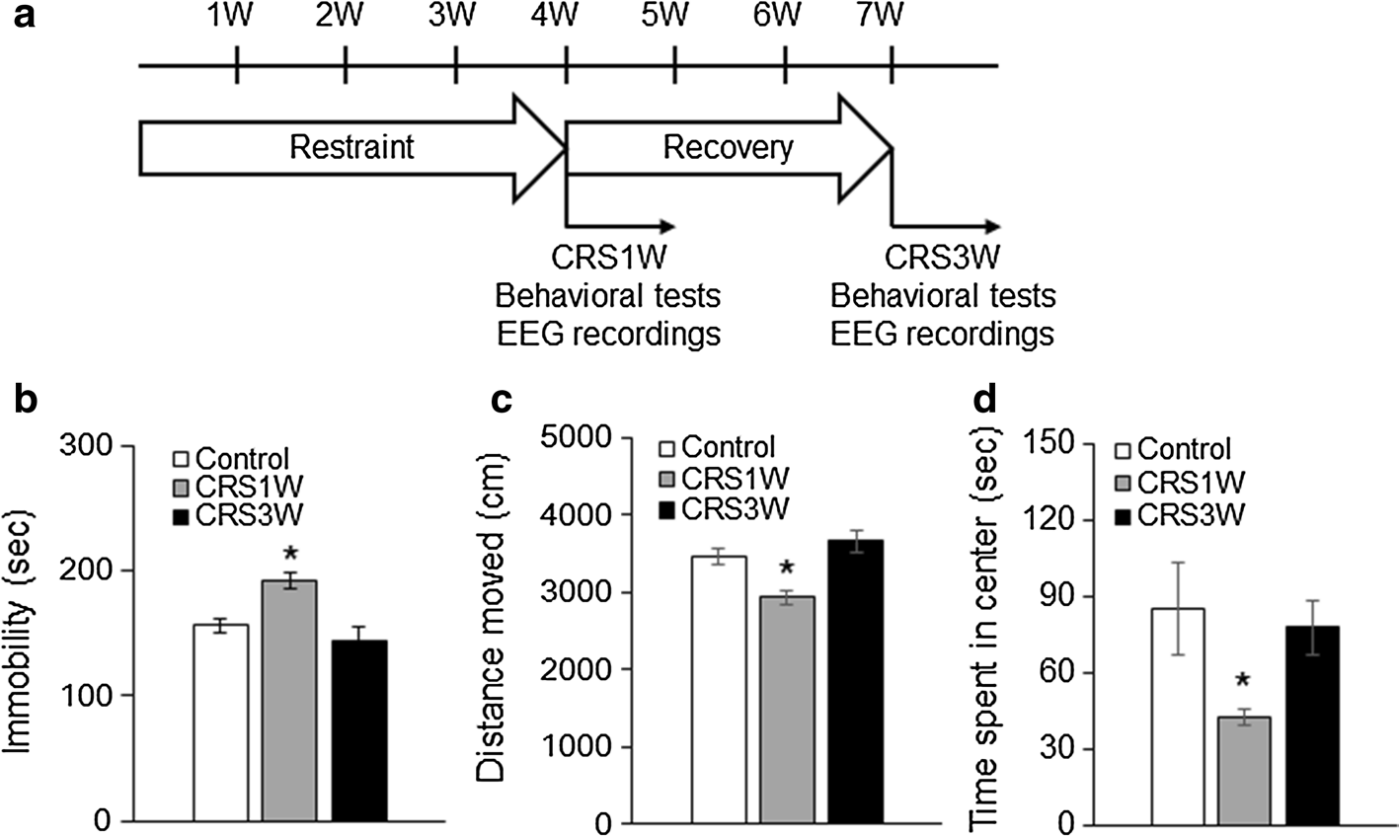
Spontaneous recovery of depression-related behaviors. **a** Time schedule of experiments, including behavioral testing and EEG recording. *W* week. **b** In FST, the CRS1W group (n = 9) showed an increase in total immobility compared to the control (n = 10) group, but the CRS3W group (n = 11) showed similar immobility to the control group. **c**, **d** In the open-field test, a decrease in the total distance moved and the time spent in the center area was observed in the CRS1W group, but the CRS3W group exhibited a normal level of locomotor activity. *P < 0.01, one-way ANOVA

We found that CRS-induced behavioral deficits spontaneously recovered during the post-CRS period, which is consistent with a previous report [16]. The forced swimming test (FST) for despair-like behavior and the open-field test for locomotor activity and anxiety were conducted at 1 week (CRS1W group) and 3 weeks (CRS3W group) following cessation of 4-week CRS (Fig. 1a). In the FST, immobility in the CRS1 W group increased compared to the control group (191.76 ± 6.27 vs. 155.82 ± 6.20 s; *p* < 0.05 by Mann–Whitney *U-*test, Fig. 1b), whereas the immobile time in the CRS3W group (144.40 ± 10.83 s) was consistent with the control group. In the open-field test, mice in the CRS1W group moved a lesser distance (2929.67 ± 115.75 vs. 3415.68 ± 113.16 s; *p* < 0.05 by Mann–Whitney *U-*test, Fig. 1c), and spent less time in the center of the open-field box (42.67 ± 3.39 vs. 84.42 ± 12.26 s; *p* < 0.05 by Mann–Whitney *U*-test, Fig. 1d) than mice in the control group. However, mice in the CRS3 W group moved a similar distance (3664.45 ± 278.77 s) (Fig. 1c), and spent a similar amount of time in the center of the box (77.65 ± 10.76 s) (Fig. 1d) than mice in the control group.

**Fig. 2:**
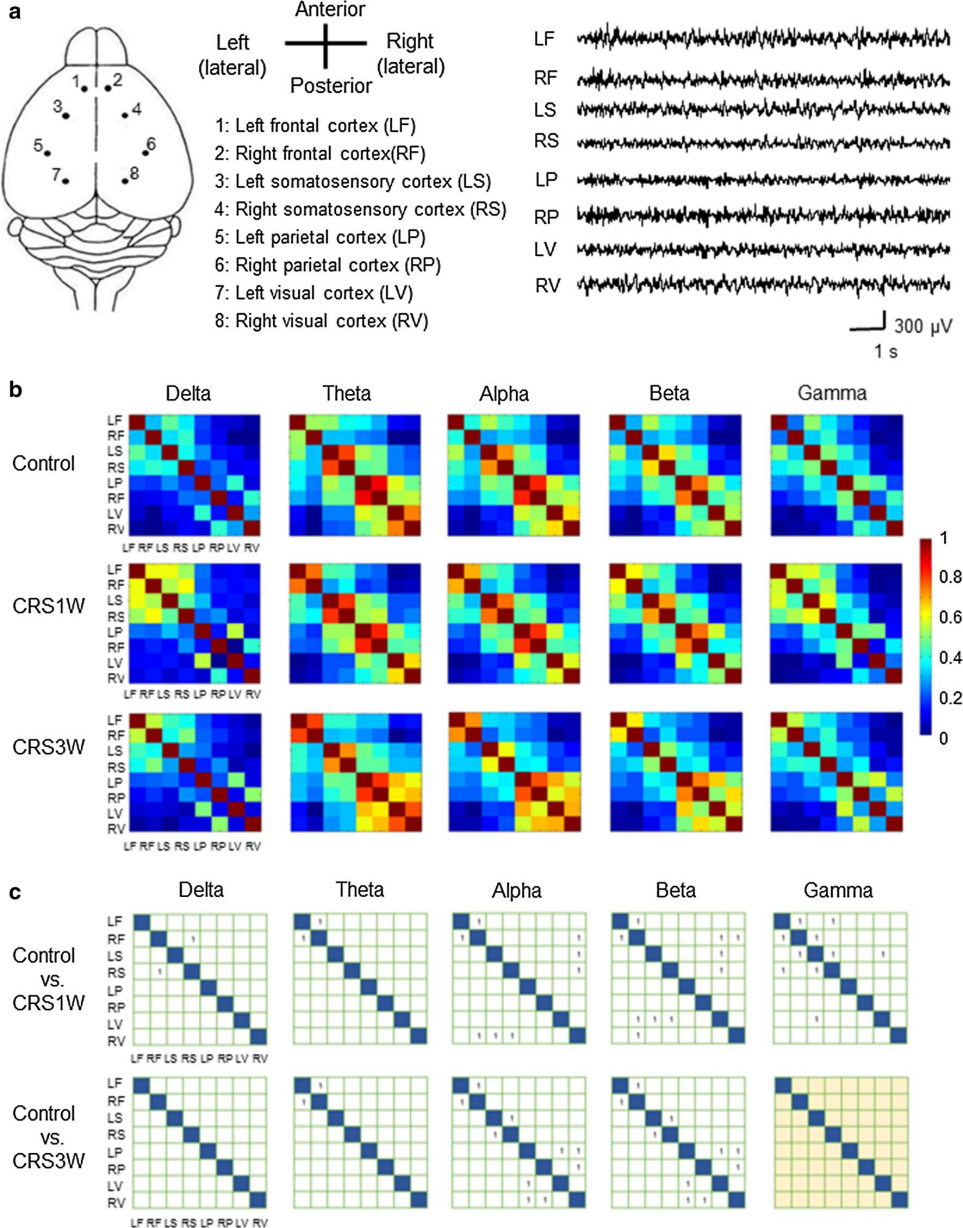
Cross-correlation analysis. **a** Drawing of a top view of the mouse brain indicating the electrode positions and names of the regions/nodes, and representative original traces of EEG recordings. **b**, **c** Cross-correlation matrices (**b**) and statistical analyses (**c**) for each pairwise correlation at five frequency-ranges, for all groups. The *colors* in (**b**) represent the strength of connectivity. Empty cells in (**c**) indicate no significant difference between the control (n = 7) and CRS1W (n = 6) or CRS3W (n = 8) groups, whereas cells with 1 indicate a significant difference among groups. Note the disappearance of the differences in the delta- and gamma-frequency bands between the control and CRS3W groups. *P* < 0.01, Kruskal–Wallis test

EEG signals were recorded from the eight cortical regions of mice in the CRS1W and CRS3W groups (Fig. 2a) to analyze regional and network brain activity. To quantify the temporal linear relationship, a cross correlation was performed between each pair of cortical regions in the CRS1W and CRS3W groups (Fig. 2b). The CRS1W group showed increased correlation coefficient values at all frequency bands in many different regions compared to the control group (Fig. 2c, Additional file 1: Table S1). However, although some of the differences in the alpha- and beta-frequency bands existed between the control and CRS3W groups, all of the differences in the delta- and gamma-frequency bands that were observed between the control and CRS1W groups could no longer be detected in the CRS3W group (Fig. 2c, Additional file 1: Table S1). These results indicate that a portion of the disrupted functional connectivity seen in the CRS1 W group returned to normal, 3 weeks following cessation of 4-week-CRS.

**Fig. 3:**
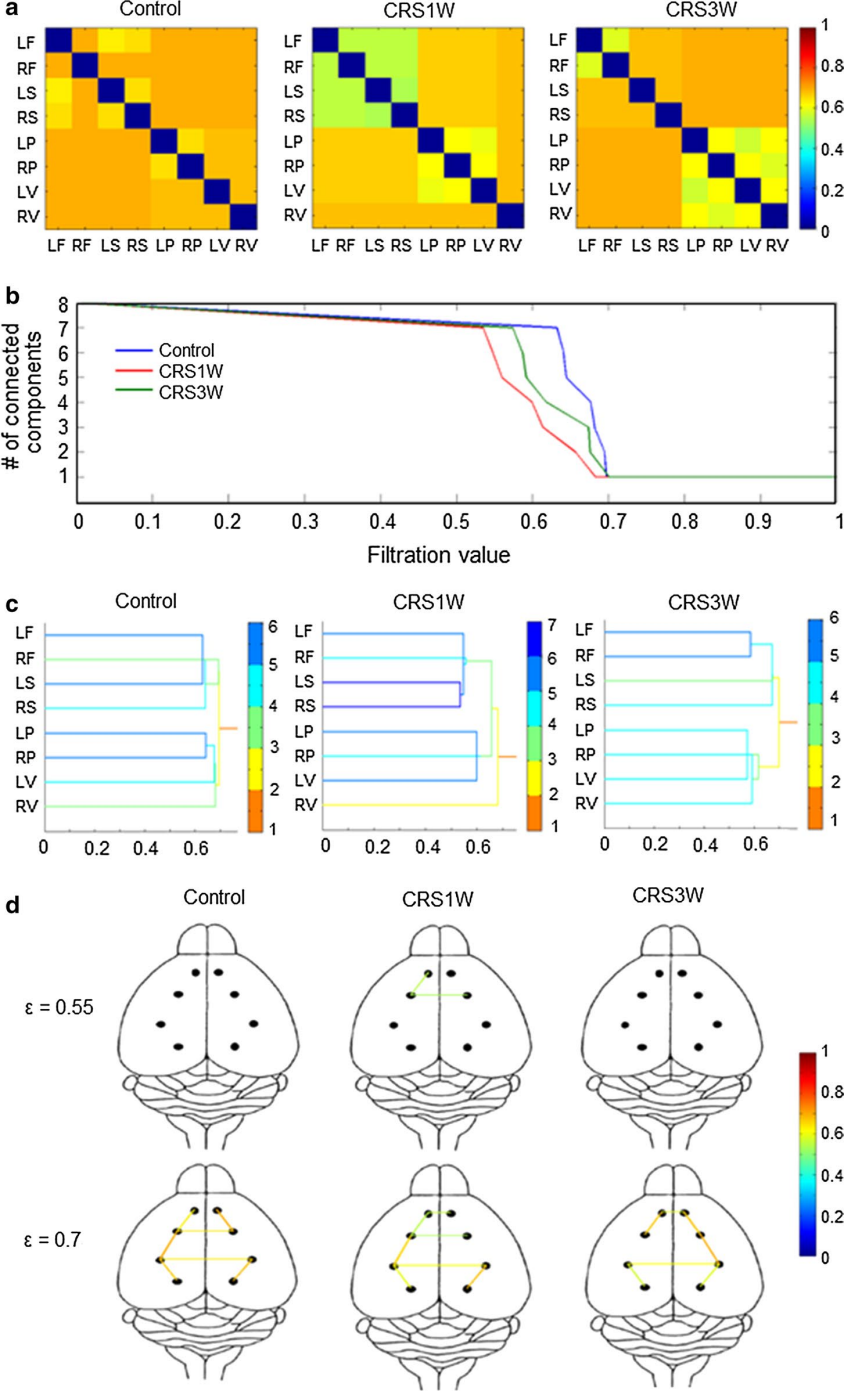
Network findings in the gamma band using persistent brain network homology. **a** Single-linkage matrices for the control, CRS1W, and CRS3W groups. The increased functional connectivity in the CRS1W group, particularly between the somatosensory and frontal cortices, compared to the control group (*P* < 0.01 corrected Bonferroni, Kruskal–Wallis test) was not evident in the CRS3W group. **b** Overlaid barcodes for each group. The CRS1 W group (ε = 0.6827) exhibited a lower final filtration value than the control group (ε = 0.6971), indicating increased global connectivity. However, the network evolution in the CRS3W group is seen to traverse to that of the control group, and a similar final filtration value was observed in the two groups (CRS3W, ε = 0.7000). **c** Dendrograms with hierarchical clustering of subnetworks. The *colors* of the *lines* represent the distance of each connected component to one final component in which all regions are connected together. Decreased functional distance between somatosensory and frontal cortices are evident in the CRS1W group, but not in the CRS3W group. **d** Brain connectivity maps at filtration values ε = 0.55 and 0.75. The *color* in the *color bar* represents the strength of functional distance. The subnetworks between the somatosensory and frontal cortices at ε = 0.55 in the CRS1W group disappeared in the CRS3W group. At ε = 0.75, the CRS1W group showed decreased functional distance (increased functional connectivity) between the somatosensory and frontal cortices, compared to the control group, and this was recovered in the CRS3W group

Next, we used a multiscale network approach, persistent brain network homology, to investigate brain connectivity at the network level. Single-linkage matrices, showing the predicted distance among the eight brain regions, were able to generate efficient separation of the brain subnetworks within each group (Additional file 2: Figure S1). The CRS1W group had decreased functional distance among many regions at different frequency bands compared to the control group, indicating increased functional connectivity in the CRS1W group (Additional file 2: Figure S2, Additional file 3: Table S2). The decreased distances in the theta-, alpha-, and beta-frequency bands in the CRS1W group did not recover in the CRS3W group. Although the decreased distances seen in the delta-frequency band in the CRS1W group recovered in the CRS3W group, newly formed increases in functional connectivity among many different regions were seen in the delta-frequency band in the CRS3W group compared to the control group. Interestingly, the CRS3W group displayed a huge restoration of the decreased distances in the gamma-frequency band (Fig. 3a, Additional file 3: Table S2).

We further examined the brain networks in the gamma-frequency band by generating barcodes for the evolution of networks and dendrograms for single-linkage hierarchical clustering, respectively. In the overlaid barcode (Fig. 3b), the control (=0.697) and CRS3W (=0.700) groups showed a similar final filtration value (i.e., maximum single-linkage distance as all components to merge in one big network), but the CRS1W group (=0.682) exhibited a lower final filtration value, indicating increased global connectivity. By constructing a dendrogram (Fig. 3c) and connectivity map (Fig. 3d), we were able to provide a visual representation of brain subnetwork change and recovery. Interestingly, the subnetworks consisting of somatosensory and frontal cortices were formed at the lower filtration values (between ε = 0.50 and ε = 0.55) in the CRS1 W group (Fig. 3c), consistent with the brain connectivity map, which featured a filtration value of ε = 0.55 (Fig. 3d). However, the control and CRS3 W groups did not show any subnetwork couplings at the same filtration-value ranges (Fig. 3c, d). In addition, at the filtration value of ε = 0.75, in which all groups showed all connections among the eight cortical regions, the CRS1W group exhibited increased strength of functional connectivity in the somatosensory and frontal cortices. However, the CRS3W group demonstrated a similar strength of connectivity among those cortices as the control group (Fig. 3d), indicating a recovery in the strength of functional connectivity. We further confirmed the recovery of functional connectivity in the CRS3 W group by analyzing another EEG epoch for stability (Additional file 4: Figure S2).

## Discussion

To date, various animal models, including chronic restraint stress, corticosterone treatment, inescapable foot-shock stress, chronic social defeat stress, and chronic unpredictable mild stress, have been established and used for understanding of the pathophysiology of depression [20]. Although not without limitations, certain depression-associated phenotypes can be reproduced independently and evaluated in mouse models. The CRS-induced depression mouse model used in this study is known to reproduce human behaviors, such as despair, anhedonia, or helplessness, which are regarded as face validity and which can be reversed by antidepressant treatment [17–19]. However, depression is a heterogeneous disorder of which the diagnostic criteria are partially subjective. Factors that affect symptom severity, along with personal treatment preferences and barriers to treatment, could also affect remission. In addition, remission rates may depend on depression severity: rates among people with severe symptoms of depression are 20–30 % lower than mild or moderate symptoms of depression [4, 5]. Thus, we cannot generalize our findings to specific clinical impacts in depression patients. To enable translation of the results, further research on the persistent brain network homology of spontaneous remission form clinical depression is essential.

## Conclusion

We were able to conclude that gamma oscillations in bilinear temporal correlations and multiscale brain networks are engaged in the spontaneous behavioral remission of depression-related behavior. Of particular interest are the gamma oscillations associated with the functional network connectivity among somatosensory and frontal cortices (Figs. 2c, 3a). Neurons participating in gamma oscillations synchronize their discharges with very high precision [21]. It has been observed that changes in gamma oscillations can give temporal integration, binding of salient stimulus features across different sensory cortices, increased spatial discreteness, and somatotopical specificity [22, 23]. Thus, gamma oscillations facilitate neuronal communication, and play a crucial role in cortical integration and perception/cognition [24]. To date, there has been no human study concerning EEG functional connectivity in relation to spontaneous behavioral remission from depression without treatment. Indeed, this is the first study in animals of the functional brain network related to spontaneous behavioral remission from depression. Our study may provide valuable insight into the pathophysiological mechanisms of depression.

## Methods

### Animals

Adult male C57BL/6 mice (7–8 weeks old) were used for the generation of chronic restraint stress (CRS)-induced mouse model of depression. All mice were housed under a 12-h light/dark cycle and had ad libitum access to food and water. Animal care and handling were carried out according to the guidelines approved by the Institutional Animal Care and Use Committee at the Korea Advanced Institute of Science and Technology.

### Generation of CRS-induced depression mouse model and behavioral task

CRS procedure was designed and conducted in line with a previous study [16]. Timeline of CRS is illustrated in Fig. 1a. For the restraint of mice, each mouse was placed in a 50 mL polystyrene tube that has evenly spaced 9 vent-holes (0.5 cm diameter each; 1.0 cm apart from each other) for 6 h, and then the restrained mice were placed in their home cage. Mice experienced restraint once a day from Day 1 to 28 (4 weeks). After 1 (CRS1W) or 3 weeks (CRS3W) (a post-CRS period) following the cessation of the 4-week-CRS, mice were subjected to behavioral tasks. Forced swimming test (FST) was used to measure behavioral despair, an indicator of depression-like behavior in mice [25, 26]. Briefly, mice were placed individually in 2000 ml glass beakers filled with nearly 1400 ml of water (10 cm from the ground, with water temperature of 25 ± 1 °C) and were allowed to swim freely for 6 min. The duration of immobility was measured during the last 4 min of the task. All behavioral tests were video-recorded. For the measurement of locomotor activity (total distance moved) and anxiety level (time in center), open-field test was performed as described previously [9, 27]. Briefly, mice were put in an open-field box made of white plastic walls (40 × 40 × 40 cm) and each mouse was placed in the periphery of the field. Then during the 10-min of video-recorded session, the total distance traveled and the time spent in the center of the box were analyzed with EthoVision (Noldus Information Technology, Wageningen, Netherlands).

### Electrode implantation and in vivo electrophysiology for EEG

EEG surgery and recording in vivo was performed as described previously [9, 27]. Animals (CRS1 W, n = 6; CRS3 W, n = 8) underwent EEG surgery on the 29th day after CRS. Animals were anesthetized by intraperitoneal injection of ketamine (90 mg/kg) and xylazine hydrochloride (40 mg/kg). Electrode implantation was performed with a stereotaxic apparatus (Kopf Instruments, Tujunga, CA, USA). EEG recordings were obtained with tungsten electrodes (0.005 in, 2 MΩ), positioned in eight cortical regions, based on a mouse brain atlas: frontal cortices (AP +1.5 mm, L ± 0.2 mm, and DV −1.0 to −1.1 mm), somatosensory cortices (AP 0.0 mm, L ± 1.5 mm, and DV −1.0 to −1.1 mm), parietal cortices (AP −2.0 mm, L ± 2.5 mm, and DV −1.0 to −1.1 mm), and visual cortices (AP −3.5 mm, L ± 1.5 mm, and DV −1.0 to −1.1 mm) in each hemisphere (Fig. 2a). A reference electrode was inserted on the skull above the cerebellum. The electrodes were fixed to the skull with cyanoacrylate adhesive and dental acrylic cement. EEG recordings of CRS1W group were performed on the 35th day, and EEG recordings of CRS3 W group were performed on the 49th day. EEG recordings were combined with video monitoring, and EEG-video recording data were obtained continuously, 2 h/day, for 2 days. EEG activity was recorded after the signal was amplified 1200-fold, band pass-filtered at 0.1-500 Hz, and digitized at a sampling rate of 1600 Hz using a digital EEG system (Comet XL, Astro-Med, West Warwick, RI, USA). The EEG-video data obtained were analyzed offline using PSG Twin (Astro-Med), Clampfit (Axon Instruments, Foster City, CA, USA), and Matlab (MathWorks, Natick, MA, USA).

### EEG analysis

Continuous EEG signals from the animals for two epochs, each consisting of 1 min of data from different days, in which they were in a resting state (i.e., awake and no movement), were analyzed to check the stability of the findings (Additional file 2: Figure S1). Then, continuous 1-min-long EEG signals from the last day of recording were used for analyses. The five EEG frequency-bands—delta (1.5–4 Hz), theta (4–8 Hz), alpha (8–12 Hz), beta (12–30 Hz), and gamma (30–80 Hz)—were analyzed for functional connectivity.

Cross-correlation: For cross-correlation analysis, the measurement set is denoted as M = {m_1_, m_2_,…, m_8_} consisting of eight nodes (i.e., eight brain regions) where we have measurement m_i_ at the ith node. We calculated cross-correlation matrix through the following equation:

$$ corr(m_{i} ,m_{j} ) = \left\langle {\frac{{m_{i} }}{{\left\| {m_{i} } \right\|}},\frac{{m_{j} }}{{\left\| {m_{j} } \right\|}}} \right\rangle $$

Pearson’s correlation coefficient was used to obtain pairwise-correlation values at zero lag.

Persistent brain network homology: We used multiscale network modelling technique known as persistent brain network homology to compare the networks of CRS models and controls effectively. Detailed procedures to quantify topological features based on persistent homology were described in a previous study [9, 27, 28]. In brief, we used networks generated at every possible threshold and to seek evolutionary changes in the subnetwork clusters by increasing the threshold in correlation matrix, which was visualized by dendrogram. The distance matrix c_M_ between two EEG measurements m_i_ and m_j_ through the following equation:

$$ c_{M} (m_{i} ,m_{j} ) = \sqrt {1 - corr(m_{i} ,m_{j} )} . $$

The brain network can be viewed as the weighted graph (M,c_M_) where M is a set of measurements at each brain region (= node) and c_M_ is the metric defined on that set. We connect the nodes i and j with an edge if the distance c_M_ (m_i_, m_j_) ≤ ε for some threshold value ε. Then the binary network B(M,ε) at threshold ε is a graph consisting of 0-simplices (nodes) and 1-simplices (edges). Start with ε = 0 and increase the ε at each iteration. The value of ε is taken discretely from the smallest c_M_(m_i_,m_j_) to largest c_M_(m_i_,m_j_). By increasing ε, more connected edges may be involved. If two nodes are already connected directly or indirectly via other intermediate nodes in smaller ε then at larger ε we don’t connect them. As a topological view of brain network, Rips complex was used to represent simplical complexes. Rips complex is defined as a simplical complex consisting of nodes and edges, whose k-simplices correspond to edges as a (k + 1)-simplices which are links of two nodes within the distance ε. Rips filtration reflects the multiscale networks, the sequence of nested Rips complexes over different scales. One of the topological features, Betti number β_0_, is a measure of the number of the connected components in the network. We could visualize those topological changes using barcode and dendrogram according to β_0_. We consider the network consisting of 0- and 1-complexes (nodes and edges). Our main concern is the changes of the zeroth Betti number β_0_, which measures the number of connected networks (CNs). The changes of β_0_ are visualized using the barcode. The vertical and horizontal axes in the barcode represent the indices of CN and filtration values respectively. The barcode of β_0_ is basically a decreasing function showing when CNs are merging to form a bigger network component. The number of CNs at the certain filtration value is same to the number of bars. If we rearrange the bars according to the node index instead of CN index in the vertical axis, we obtain single linkage dendrogram (SLD). While the barcode of β_0_ shows the global changes of the connected structure of network when the bars are ended, the SLD shows the local changes when the bars are merged. SLD between the nodes was calculated, which is usually used in hierarchical clustering. Given the network with distance c_M_, we calculated SLD (d_M_), which was defines as:

$$ d_{M} (m_{i} ,m_{j} ) = \hbox{min} \{ \mathop {\hbox{max} }\limits_{l = 0, \ldots ,k - 1} C_{M} (w_{l} ,w_{l = 1} )/m_{i} = w_{0} , \ldots , w_{k} = m_{j} \} $$

where m_i_ = w_0_,…,w_k_ = m_j_ be a path between m_i_ and m_j_. SLD is the minimum distance between two nodes when they belong to the same connected component during Rips filtration. It represents the hierarchical clustered structure of brain network in an algebraic form which can be used for a quantitative measure to discriminate brain networks. Using SLD calculated from persistent network homology, we could obtain the distance between two nodes after network construction without specific threshold. Each entry in the single-linkage matrix is a model-based predicted functional distance between the two nodes, m_i_ and m_j_. The model-predicted distances from single-linkage matrices were tested with the Kruskal–Wallis test at the 0.05 level of significance with a Bonferroni correction.

### Statistical analyses

Data collected were expressed as mean ± standard error of the mean (S.E.M.). For intergroup comparisons of behaviors, a one-way ANOVA or Mann–Whitney U test was used for comparisons among groups or between dependent variables. A *P* value <0.05 was considered significant. Kruskal–Wallis test was performed for the statistical comparison of slopes and final filtration values of the barcodes between the groups. Kruskal–Wallis test was used to compare pairwise single linkage matrices with a Bonferroni correction. SPSS 21.0 (SPSS Inc, Chicago, Ill, USA) and Matlab were used for the statistical analyses.

## Additional files

10.1186/s12868-016-0239-x Statistical results of the cross-correlation in Fig. 2c.
10.1186/s12868-016-0239-x Single-linkage matrices showing the predicted distances among eight brain regions in each group. The CRS1W and CRS3W groups showed decreased functional distance (i.e., increased functional connectivity) between many regions in the delta-, theta-, alpha-, and beta-frequency bands compared to the control group (*p* < 0.05, corrected Bonferroni, Kruskal–Wallis test).
10.1186/s12868-016-0239-x Statistical results of the single-linkage matrices in Fig. 3a and Additional file 2: Figure S1.
10.1186/s12868-016-0239-x Stability test using two different epochs of EEG signals to confirm network analysis results. (A) The absolute difference matrix between cross-correlation values obtained from two epochs selected at different time points (i.e., two different epochs). The difference at the main diagonal of the box is very small or almost zero, indicating that the EEG data are stable. (B) Barcodes for both extracted epochs in the gamma-frequency band show a similar pattern: the shape of the barcode and the final filtration value in the CRS3 W group is more similar to that of the control group than the CRS1 W group.

## Abbreviations

CRS: chronic restraint stress
EEG: electroencephalography
FST: forced swimming test
SLD: single linkage dendrogram
CNs: connected networks
