# Supplementary material for: Gamma oscillation in functional brain networks is involved in the spontaneous remission of depressive behavior induced by chronic restraint stress in mice
Source: BMC Neurosci. 2016 Jan 12;17:4. doi: 10.1186/s12868-016-0239-x (PMC4710024; doi:10.1186/s12868-016-0239-x)
Supplement: Supplementary file 1 — 10.1186/s12868-016-0239-x Statistical results of the cross-correlation in Fig. 2c. [file 12868_2016_239_MOESM1_ESM.pdf]

Supplementary Table 1. Statistical results of cross-correlation in Figure 2c. (Intercation between groups)

Recovery is indicated by yellow-colored box

New difference is indicated by red-colored box

Delta	1, Control; 2, CRS1W; 3, CRS3W		95% confidence interval for the true mean difference (Lower bound)	Mean difference between the groups	95% confidence interval for the true mean difference (Upper bound)	p-value	Decision (1 = Significant difference, 0 = Not Significant)
LF/RF	1	2	-12.8524823	-4.761904762	3.328672776	0.3517	0
	1	3	-10.32991621	-2.803571429	4.722773353	0.6573	0
LF/LS	1	2	-12.61438706	-4.523809524	3.566768014	0.3893	0
	1	3	-3.758487639	3.767857143	11.29420192	0.4692	0
LF/RS	1	2	-13.40010135	-5.30952381	2.781053728	0.2731	0
	1	3	-6.544201925	0.982142857	8.508487639	0.9497	0
LF/LP	1	2	-6.3524823	1.738095238	9.828672776	0.8696	0
	1	3	-4.70491621	2.821428571	10.34777335	0.6538	0
LF/RP	1	2	-7.614387062	0.476190476	8.566768014	0.9896	0
	1	3	-7.508487639	0.017857143	7.544201925	1	0
LF/LV	1	2	-6.733434681	1.357142857	9.447720395	0.9183	0
	1	3	-3.669201925	3.857142857	11.38348764	0.4525	0
LF/RV	1	2	-6.638196585	1.452380952	9.54295849	0.9071	0
	1	3	-2.615630496	4.910714286	12.43705907	0.2772	0
RF/LS	1	2	-13.54295849	-5.452380952	2.638196585	0.2545	0
	1	3	-6.812059068	0.714285714	8.240630496	0.9731	0
RF/RS	1	2	-16.30486325	-8.214285714	-0.123708176	0.0456	1
	1	3	-8.490630496	-0.964285714	6.562059068	0.9515	0
RF/LP	1	2	-7.947720395	0.142857143	8.233434681	0.9991	0
	1	3	-7.258487639	0.267857143	7.794201925	0.9962	0
RF/RP	1	2	-6.995339443	1.095238095	9.185815633	0.946	0
	1	3	-7.222773353	0.303571429	7.82991621	0.9951	0
RF/LV	1	2	-8.947720395	-0.857142857	7.233434681	0.9666	0
	1	3	-6.508487639	1.017857143	8.544201925	0.9461	0
RF/RV	1	2	-7.304863252	0.785714286	8.876291824	0.9718	0
	1	3	-4.740630496	2.785714286	10.31205907	0.6609	0
LS/RS	1	2	-11.13819659	-3.047619048	5.04295849	0.6512	0
	1	3	-1.865630496	5.660714286	13.18705907	0.1823	0
LS/LP	1	2	-7.376291824	0.714285714	8.804863252	0.9767	0
	1	3	-3.562059068	3.964285714	11.4906305	0.4328	0
LS/RP	1	2	-6.328672776	1.761904762	9.8524823	0.8663	0
	1	3	-2.472773353	5.053571429	12.57991621	0.2571	0
LS/LV	1	2	-7.709625157	0.380952381	8.471529919	0.9933	0
	1	3	-3.312059068	4.214285714	11.7406305	0.3882	0
LS/RV	1	2	-6.733434681	1.357142857	9.447720395	0.9183	0
	1	3	-1.044201925	6.482142857	14.00848764	0.1077	0
RS/LP	1	2	-7.209625157	0.880952381	8.971529919	0.9647	0
	1	3	-3.687059068	3.839285714	11.3656305	0.4558	0
RS/RP	1	2	-7.781053728	0.30952381	8.400101347	0.9956	0
	1	3	-7.383487639	0.142857143	7.669201925	0.9989	0
RS/LV	1	2	-5.114387062	2.976190476	11.06676801	0.6642	0
	1	3	-1.508487639	6.017857143	13.54420192	0.1463	0
RS/RV	1	2	-4.04295849	4.047619048	12.13819659	0.4696	0
	1	3	-3.437059068	4.089285714	11.6156305	0.4102	0
LP/RP	1	2	-4.685815633	3.404761905	11.49533944	0.5855	0
	1	3	-5.95491621	1.571428571	9.097773353	0.8764	0

LP/LV	1	2	-13.04295849	-4.952380952	3.138196585	0.3231	0
	1	3	-9.812059068	-2.285714286	5.240630496	0.7565	0
LP/RV	1	2	-4.781053728	3.30952381	11.40010135	0.603	0
	1	3	-4.383487639	3.142857143	10.66920192	0.5904	0
RP/LV	1	2	-1.114387062	6.976190476	15.06676801	0.1072	0
	1	3	-4.508487639	3.017857143	10.54420192	0.6151	0
RP/RV	1	2	-8.781053728	-0.69047619	7.400101347	0.9782	0
	1	3	-9.258487639	-1.732142857	5.794201925	0.8519	0
LV/RV	1	2	-2.423910871	5.666666667	13.7572442	0.2282	0
	1	3	-1.276344782	6.25	13.77634478	0.1259	0

**Theta**	**1, Control; 2, CRS1W; 3, CRS3W**		**95% confidence interval for the true mean difference (Lower bound)**	**Mean difference between the groups**	**95% confidence interval for the true mean difference (Upper bound)**	**p-value**	**Decision (1 = Significant difference, 0 = Not Significant)**
LF/RF	1	2	-16.37629182	-8.285714286	-0.195136748	0.0433	1
	1	3	-15.18705907	-7.660714286	-0.134369504	0.0449	1
LF/LS	1	2	-8.090577538	0	8.090577538	1	0
	1	3	-2.276344782	5.25	12.77634478	0.231	0
LF/RS	1	2	-9.590577538	-1.5	6.590577538	0.9012	0
	1	3	-6.401344782	1.125	8.651344782	0.9346	0
LF/LP	1	2	-3.019148966	5.071428571	13.16200611	0.3059	0
	1	3	-1.95491621	5.571428571	13.09777335	0.1922	0
LF/RP	1	2	-6.257244205	1.833333333	9.923910871	0.8561	0
	1	3	-3.651344782	3.875	11.40134478	0.4492	0
LF/LV	1	2	-6.162006109	1.928571429	10.01914897	0.842	0
	1	3	-5.222773353	2.303571429	9.82991621	0.7532	0
LF/RV	1	2	-6.519148966	1.571428571	9.662006109	0.8921	0
	1	3	-4.57991621	2.946428571	10.47277335	0.6292	0
RF/LS	1	2	-7.638196585	0.452380952	8.54295849	0.9906	0
	1	3	-4.490630496	3.035714286	10.56205907	0.6115	0
RF/RS	1	2	-9.662006109	-1.571428571	6.519148966	0.8921	0
	1	3	-5.222773353	2.303571429	9.82991621	0.7532	0
RF/LP	1	2	-5.614387062	2.476190476	10.56676801	0.7532	0
	1	3	-3.758487639	3.767857143	11.29420192	0.4692	0
RF/RP	1	2	-7.423910871	0.666666667	8.757244205	0.9796	0
	1	3	-2.776344782	4.75	12.27634478	0.3009	0
RF/LV	1	2	-9.162006109	-1.071428571	7.019148966	0.9483	0
	1	3	-2.972773353	4.553571429	12.07991621	0.3316	0
RF/RV	1	2	-10.61438706	-2.523809524	5.566768014	0.745	0
	1	3	-5.258487639	2.267857143	9.794201925	0.7598	0
LS/RS	1	2	-12.56676801	-4.476190476	3.614387062	0.397	0
	1	3	-1.919201925	5.607142857	13.13348764	0.1882	0
LS/LP	1	2	-9.066768014	-0.976190476	7.114387062	0.9569	0
	1	3	-1.919201925	5.607142857	13.13348764	0.1882	0
LS/RP	1	2	-8.876291824	-0.785714286	7.304863252	0.9718	0
	1	3	-2.437059068	5.089285714	12.6156305	0.2522	0
LS/LV	1	2	-7.590577538	0.5	8.590577538	0.9885	0
	1	3	-2.651344782	4.875	12.40134478	0.2824	0
LS/RV	1	2	-9.685815633	-1.595238095	6.495339443	0.889	0
	1	3	-4.82991621	2.696428571	10.22277335	0.6783	0
RS/LP	1	2	-11.16200611	-3.071428571	5.019148966	0.6468	0
	1	3	-4.097773353	3.428571429	10.95491621	0.5342	0
RS/RP	1	2	-13.61438706	-5.523809524	2.566768014	0.2455	0

	1	3	-5.633487639	1.892857143	9.419201925	0.8258	0
RS/LV	1	2	-7.757244205	0.333333333	8.423910871	0.9949	0
	1	3	-5.151344782	2.375	9.901344782	0.7399	0
RS/RV	1	2	-11.66200611	-3.571428571	4.519148966	0.555	0
	1	3	-6.347773353	1.178571429	8.70491621	0.9284	0
LP/RP	1	2	-5.685815633	2.404761905	10.49533944	0.7655	0
	1	3	-2.57991621	4.946428571	12.47277335	0.2721	0
LP/LV	1	2	-10.2572442	-2.166666667	5.923910871	0.8049	0
	1	3	-11.15134478	-3.625	3.901344782	0.4963	0
LP/RV	1	2	-10.59057754	-2.5	5.590577538	0.7491	0
	1	3	-10.90134478	-3.375	4.151344782	0.5447	0
RP/LV	1	2	-8.328672776	-0.238095238	7.8524823	0.9974	0
	1	3	-11.47277335	-3.946428571	3.57991621	0.436	0
RP/RV	1	2	-11.61438706	-3.523809524	4.566768014	0.5637	0
	1	3	-12.38348764	-4.857142857	2.669201925	0.285	0
LV/RV	1	2	-5.685815633	2.404761905	10.49533944	0.7655	0
	1	3	-7.82991621	-0.303571429	7.222773353	0.9951	0

**Alpha**	**1, Control; 2, CRS1W; 3, CRS3W**		**95% confidence interval for the true mean difference (Lower bound)**	**Mean difference between the groups**	**95% confidence interval for the true mean difference (Upper bound)**	**p-value**	**Decision (1 = Significant difference, 0 = Not Significant)**
LF/RF	1	2	-17.28105373	-9.19047619	-1.099898653	0.0212	1
	1	3	-16.00848764	-8.482142857	-0.955798075	0.0225	1
LF/LS	1	2	-8.281053728	-0.19047619	7.900101347	0.9983	0
	1	3	-1.758487639	5.767857143	13.29420192	0.1709	0
LF/RS	1	2	-9.400101347	-1.30952381	6.781053728	0.9237	0
	1	3	-4.294201925	3.232142857	10.75848764	0.5727	0
LF/LP	1	2	-6.04295849	2.047619048	10.13819659	0.8238	0
	1	3	-4.562059068	2.964285714	10.4906305	0.6257	0
LF/RP	1	2	-8.781053728	-0.69047619	7.400101347	0.9782	0
	1	3	-6.633487639	0.892857143	8.419201925	0.9583	0
LF/LV	1	2	-10.61438706	-2.523809524	5.566768014	0.745	0
	1	3	-7.883487639	-0.357142857	7.169201925	0.9932	0
LF/RV	1	2	-12.30486325	-4.214285714	3.876291824	0.4408	0
	1	3	-8.865630496	-1.339285714	6.187059068	0.9086	0
RF/LS	1	2	-11.92391087	-3.833333333	4.257244205	0.5076	0
	1	3	-7.276344782	0.25	7.776344782	0.9967	0
RF/RS	1	2	-11.92391087	-3.833333333	4.257244205	0.5076	0
	1	3	-7.276344782	0.25	7.776344782	0.9967	0
RF/LP	1	2	-10.51914897	-2.428571429	5.662006109	0.7614	0
	1	3	-6.82991621	0.696428571	8.222773353	0.9744	0
RF/RP	1	2	-12.04295849	-3.952380952	4.138196585	0.4864	0
	1	3	-7.937059068	-0.410714286	7.115630496	0.991	0
RF/LV	1	2	-14.8524823	-6.761904762	1.328672776	0.1226	0
	1	3	-8.82991621	-1.303571429	6.222773353	0.9132	0
RF/RV	1	2	-16.56676801	-8.476190476	-0.385612938	0.0374	1
	1	3	-12.04420192	-4.517857143	3.008487639	0.3373	0
LS/RS	1	2	-12.82867278	-4.738095238	3.3524823	0.3554	0
	1	3	-0.222773353	7.303571429	14.82991621	0.0594	1
LS/LP	1	2	-10.01914897	-1.928571429	6.162006109	0.842	0
	1	3	-1.95491621	5.571428571	13.09777335	0.1922	0
LS/RP	1	2	-12.28105373	-4.19047619	3.900101347	0.4449	0
	1	3	-4.008487639	3.517857143	11.04420192	0.5169	0

LS/LV	1	2	-14.42391087	-6.333333333	1.757244205	0.1583	0
	1	3	-10.65134478	-3.125	4.401344782	0.5939	0
LS/RV	1	2	-16.32867278	-8.238095238	-0.1475177	0.0448	1
	1	3	-10.72277335	-3.196428571	4.32991621	0.5798	0
RS/LP	1	2	-10.68581563	-2.595238095	5.495339443	0.7325	0
	1	3	-4.07991621	3.446428571	10.97277335	0.5307	0
RS/RP	1	2	-12.7572442	-4.666666667	3.423910871	0.3665	0
	1	3	-6.651344782	0.875	8.401344782	0.9599	0
RS/LV	1	2	-15.61438706	-7.523809524	0.566768014	0.0747	0
	1	3	-12.00848764	-4.482142857	3.044201925	0.3431	0
RS/RV	1	2	-17.40010135	-9.30952381	-1.218946272	0.0192	1
	1	3	-8.794201925	-1.267857143	6.258487639	0.9177	0
LP/RP	1	2	-10.47152992	-2.380952381	5.709625157	0.7695	0
	1	3	-4.990630496	2.535714286	10.06205907	0.7094	0
LP/LV	1	2	-15.51914897	-7.428571429	0.662006109	0.0797	0
	1	3	-16.20491621	-8.678571429	-1.152226647	0.0189	1
LP/RV	1	2	-15.59057754	-7.5	0.590577538	0.0759	0
	1	3	-15.02634478	-7.5	0.026344782	0.051	1
RP/LV	1	2	-11.44772039	-3.357142857	4.733434681	0.5943	0
	1	3	-12.50848764	-4.982142857	2.544201925	0.267	0
RP/RV	1	2	-14.49533944	-6.404761905	1.685815633	0.1519	0
	1	3	-14.72277335	-7.196428571	0.32991621	0.0645	1
LV/RV	1	2	-11.8524823	-3.761904762	4.328672776	0.5204	0
	1	3	-11.07991621	-3.553571429	3.972773353	0.51	0

**Beta**	**1, Control; 2, CRS1W; 3, CRS3W**		**95% confidence interval for the true mean difference (Lower bound)**	**Mean difference between the groups**	**95% confidence interval for the true mean difference (Upper bound)**	**p-value**	**Decision (1 = Significant difference, 0 = Not Significant)**
LF/RF	1	2	-17.28105373	-9.19047619	-1.099898653	0.0212	1
	1	3	-16.00848764	-8.482142857	-0.955798075	0.0225	1
LF/LS	1	2	-11.09057754	-3	5.090577538	0.6599	0
	1	3	-2.651344782	4.875	12.40134478	0.2824	0
LF/RS	1	2	-11.90010135	-3.80952381	4.281053728	0.5119	0
	1	3	-5.044201925	2.482142857	10.00848764	0.7197	0
LF/LP	1	2	-9.876291824	-1.785714286	6.304863252	0.8629	0
	1	3	-6.937059068	0.589285714	8.115630496	0.9816	0
LF/RP	1	2	-12.66200611	-4.571428571	3.519148966	0.3816	0
	1	3	-10.84777335	-3.321428571	4.20491621	0.5552	0
LF/LV	1	2	-14.11438706	-6.023809524	2.066768014	0.1885	0
	1	3	-10.50848764	-2.982142857	4.544201925	0.6221	0
LF/RV	1	2	-13.94772039	-5.857142857	2.233434681	0.2064	0
	1	3	-10.63348764	-3.107142857	4.419201925	0.5974	0
RF/LS	1	2	-12.70962516	-4.619047619	3.471529919	0.374	0
	1	3	-7.437059068	0.089285714	7.615630496	0.9996	0
RF/RS	1	2	-12.82867278	-4.738095238	3.3524823	0.3554	0
	1	3	-8.097773353	-0.571428571	6.95491621	0.9827	0
RF/LP	1	2	-11.2572442	-3.166666667	4.923910871	0.6293	0
	1	3	-7.776344782	-0.25	7.276344782	0.9967	0
RF/RP	1	2	-13.47152992	-5.380952381	2.709625157	0.2637	0
	1	3	-10.6156305	-3.089285714	4.437059068	0.601	0
RF/LV	1	2	-16.7572442	-8.666666667	-0.576089129	0.0323	1
	1	3	-11.52634478	-4	3.526344782	0.4263	0
RF/RV	1	2	-17.61438706	-9.523809524	-1.433231986	0.016	1

	1	3	-13.13348764	-5.607142857	1.919201925	0.1882	0
LS/RS	1	2	-13.59057754	-5.5	2.590577538	0.2485	0
	1	3	-0.776344782	6.75	14.27634478	0.0894	1
LS/LP	1	2	-10.2572442	-2.166666667	5.923910871	0.8049	0
	1	3	-3.276344782	4.25	11.77634478	0.3821	0
LS/RP	1	2	-12.68581563	-4.595238095	3.495339443	0.3778	0
	1	3	-5.20491621	2.321428571	9.847773353	0.7499	0
LS/LV	1	2	-16.37629182	-8.285714286	-0.195136748	0.0433	1
	1	3	-12.56205907	-5.035714286	2.490630496	0.2595	0
LS/RV	1	2	-16.92391087	-8.833333333	-0.742755795	0.0283	0
	1	3	-11.40134478	-3.875	3.651344782	0.4492	0
RS/LP	1	2	-11.44772039	-3.357142857	4.733434681	0.5943	0
	1	3	-4.633487639	2.892857143	10.41920192	0.6398	0
RS/RP	1	2	-12.92391087	-4.833333333	3.257244205	0.3408	0
	1	3	-6.526344782	1	8.526344782	0.948	0
RS/LV	1	2	-15.92391087	-7.833333333	0.257244205	0.0602	1
	1	3	-12.15134478	-4.625	2.901344782	0.3202	0
RS/RV	1	2	-15.97152992	-7.880952381	0.209625157	0.0582	0
	1	3	-11.3656305	-3.839285714	3.687059068	0.4558	0
LP/RP	1	2	-13.04295849	-4.952380952	3.138196585	0.3231	0
	1	3	-7.187059068	0.339285714	7.865630496	0.9939	0
LP/LV	1	2	-15.32867278	-7.238095238	0.8524823	0.0905	0
	1	3	-16.72277335	-9.196428571	-1.67008379	0.0117	1
LP/RV	1	2	-15.40010135	-7.30952381	0.781053728	0.0863	0
	1	3	-15.54420192	-8.017857143	-0.491512361	0.0335	1
RP/LV	1	2	-13.63819659	-5.547619048	2.54295849	0.2426	0
	1	3	-13.1156305	-5.589285714	1.937059068	0.1902	0
RP/RV	1	2	-15.2572442	-7.166666667	0.923910871	0.0948	0
	1	3	-15.27634478	-7.75	-0.223655218	0.0418	1
LV/RV	1	2	-12.51914897	-4.428571429	3.662006109	0.4048	0
	1	3	-10.57991621	-3.053571429	4.472773353	0.608	0

**Gamm**	**1, Control; 2, CRS1W; 3, CRS3W**		**95% confidence interval for the true mean difference (Lower bound)**	**Mean difference between the groups**	**95% confidence interval for the true mean difference (Upper bound)**	**p-value**	**Decision (1 = Significant difference, 0 = Not Significant)**
LF/RF	1	2	-16.90010135	-8.80952381	-0.718946272	0.0289	1
	1	3	-14.41920192	-6.892857143	0.633487639	0.0807	0
LF/LS	1	2	-13.73343468	-5.642857143	2.447720395	0.231	0
	1	3	-3.669201925	3.857142857	11.38348764	0.4525	0
LF/RS	1	2	-18.30486325	-10.21428571	-2.123708176	0.0087	1
	1	3	-9.615630496	-2.089285714	5.437059068	0.792	0
LF/LP	1	2	-11.06676801	-2.976190476	5.114387062	0.6642	0
	1	3	-8.294201925	-0.767857143	6.758487639	0.969	0
LF/RP	1	2	-14.42391087	-6.333333333	1.757244205	0.1583	0
	1	3	-13.27634478	-5.75	1.776344782	0.1727	0
LF/LV	1	2	-14.06676801	-5.976190476	2.114387062	0.1935	0
	1	3	-11.29420192	-3.767857143	3.758487639	0.4692	0
LF/RV	1	2	-12.73343468	-4.642857143	3.447720395	0.3703	0
	1	3	-9.669201925	-2.142857143	5.383487639	0.7825	0
RF/LS	1	2	-16.73343468	-8.642857143	-0.552279605	0.0329	1
	1	3	-6.669201925	0.857142857	8.383487639	0.9615	0
RF/RS	1	2	-14.70962516	-6.619047619	1.471529919	0.1337	0
	1	3	-8.562059068	-1.035714286	6.490630496	0.9443	0

RF/LP	1	2	-12.44772039	-4.357142857	3.733434681	0.4167	0
	1	3	-9.133487639	-1.607142857	5.919201925	0.8711	0
RF/RP	1	2	-13.18581563	-5.095238095	2.995339443	0.3025	0
	1	3	-12.70491621	-5.178571429	2.347773353	0.2402	0
RF/LV	1	2	-14.13819659	-6.047619048	2.04295849	0.1861	0
	1	3	-10.1156305	-2.589285714	4.937059068	0.6991	0
RF/RV	1	2	-13.63819659	-5.547619048	2.54295849	0.2426	0
	1	3	-10.4906305	-2.964285714	4.562059068	0.6257	0
LS/RS	1	2	-16.30486325	-8.214285714	-0.123708176	0.0456	1
	1	3	-3.240630496	4.285714286	11.81205907	0.3759	0
LS/LP	1	2	-11.23343468	-3.142857143	4.947720395	0.6337	0
	1	3	-5.544201925	1.982142857	9.508487639	0.8107	0
LS/RP	1	2	-13.49533944	-5.404761905	2.685815633	0.2606	0
	1	3	-7.597773353	-0.071428571	7.45491621	0.9997	0
LS/LV	1	2	-15.66200611	-7.571428571	0.519148966	0.0723	1
	1	3	-11.22277335	-3.696428571	3.82991621	0.4826	0
LS/RV	1	2	-13.78105373	-5.69047619	2.400101347	0.2254	0
	1	3	-8.133487639	-0.607142857	6.919201925	0.9805	0
RS/LP	1	2	-12.11438706	-4.023809524	4.066768014	0.4738	0
	1	3	-6.758487639	0.767857143	8.294201925	0.969	0
RS/RP	1	2	-14.59057754	-6.5	1.590577538	0.1436	0
	1	3	-10.52634478	-3	4.526344782	0.6186	0
RS/LV	1	2	-15.09057754	-7	1.090577538	0.1056	0
	1	3	-10.15134478	-2.625	4.901344782	0.6922	0
RS/RV	1	2	-13.51914897	-5.428571429	2.662006109	0.2576	0
	1	3	-9.82991621	-2.303571429	5.222773353	0.7532	0
LP/RP	1	2	-10.28105373	-2.19047619	5.900101347	0.8011	0
	1	3	-10.75848764	-3.232142857	4.294201925	0.5727	0
LP/LV	1	2	-14.54295849	-6.452380952	1.638196585	0.1477	0
	1	3	-16.56205907	-9.035714286	-1.509369504	0.0836	0
LP/RV	1	2	-10.66200611	-2.571428571	5.519148966	0.7367	0
	1	3	-12.34777335	-4.821428571	2.70491621	0.2902	0
RP/LV	1	2	-7.685815633	0.404761905	8.495339443	0.9924	0
	1	3	-11.57991621	-4.053571429	3.472773353	0.4166	0
RP/RV	1	2	-13.54295849	-5.452380952	2.638196585	0.2545	0
	1	3	-14.68705907	-7.160714286	0.365630496	0.0662	0
LV/RV	1	2	-8.04295849	0.047619048	8.138196585	0.9999	0
	1	3	-5.687059068	1.839285714	9.365630496	0.8347	0
