# Supplementary figures and images for: Gamma oscillation in functional brain networks is involved in the spontaneous remission of depressive behavior induced by chronic restraint stress in mice
Source: BMC Neurosci. 2016 Jan 12;17:4. doi: 10.1186/s12868-016-0239-x (PMC4710024; doi:10.1186/s12868-016-0239-x)

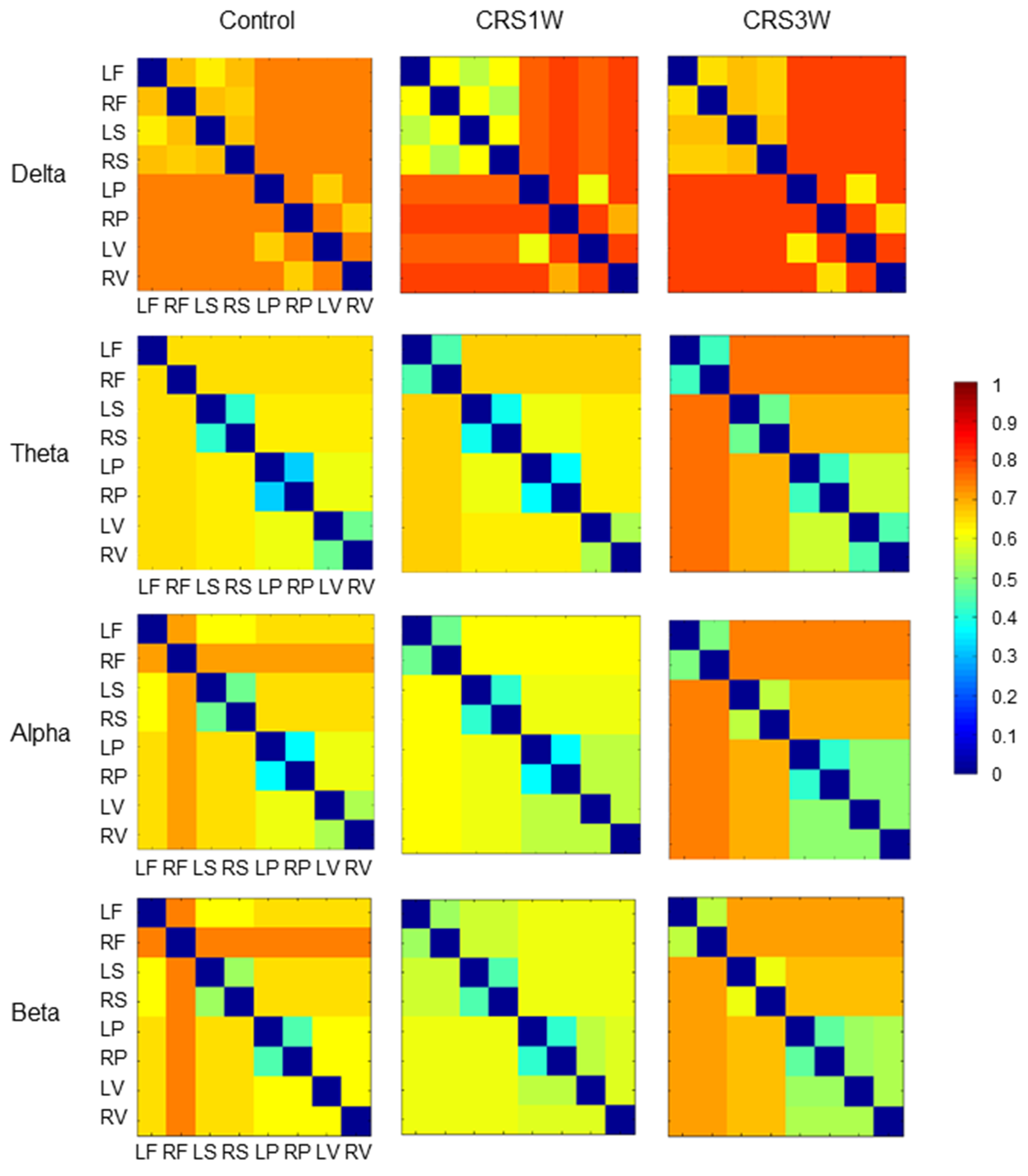

Supplement: Supplementary file 2 — 10.1186/s12868-016-0239-x Single-linkage matrices showing the predicted distances among eight brain regions in each group. The CRS1W and CRS3W groups showed decreased functional distance (i.e., increased functional connectivity) between many regions in the delta-, theta-, alpha-, and beta-frequency bands compared to the control group (p < 0.05, corrected Bonferroni, Kruskal–Wallis test). [file 12868_2016_239_MOESM2_ESM.tiff]

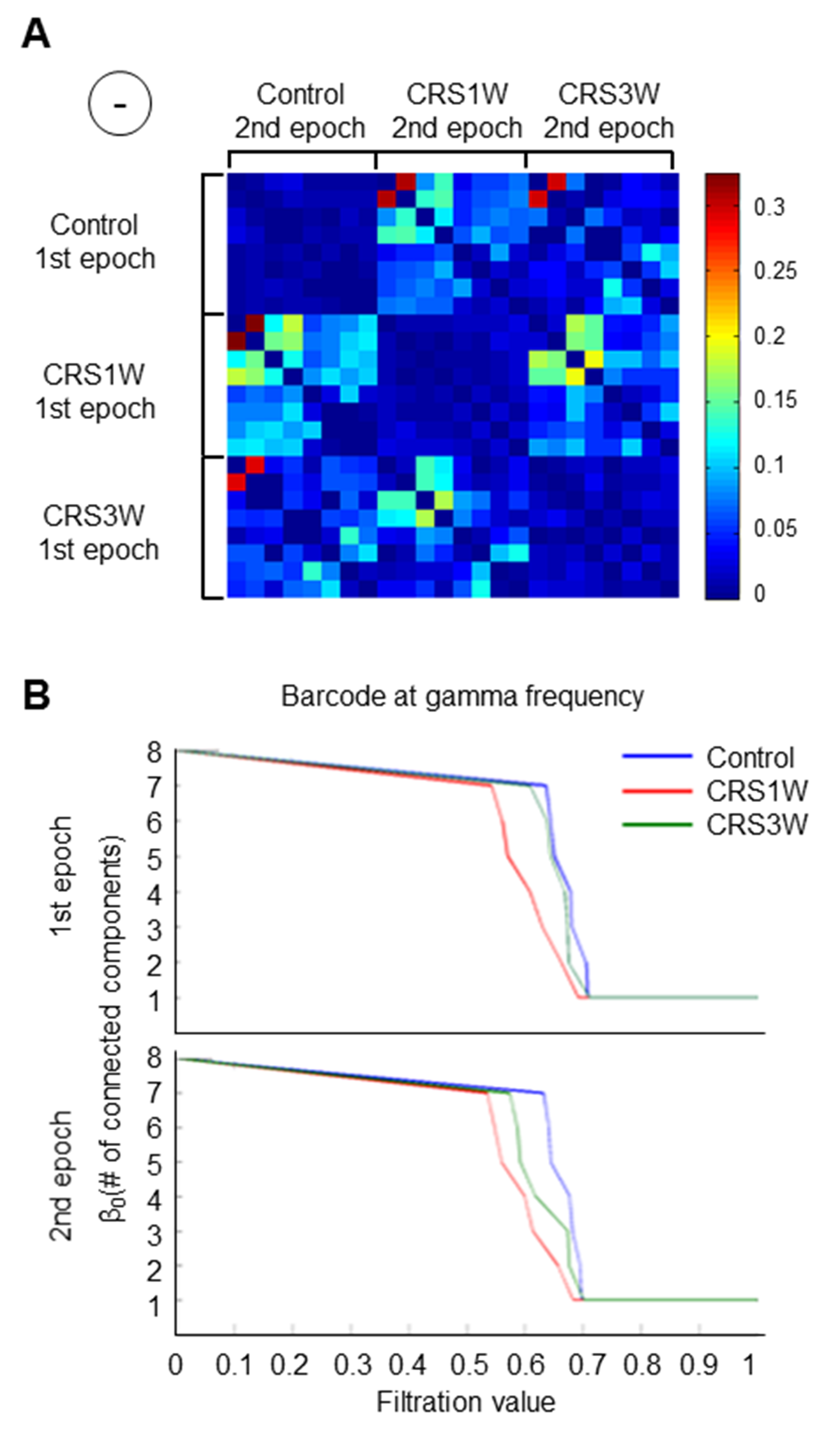

Supplement: Supplementary file 4 — 10.1186/s12868-016-0239-x Stability test using two different epochs of EEG signals to confirm network analysis results. (A) The absolute difference matrix between cross-correlation values obtained from two epochs selected at different time points (i.e., two different epochs). The difference at the main diagonal of the box is very small or almost zero, indicating that the EEG data are stable. (B) Barcodes for both extracted epochs in the gamma-frequency band show a similar pattern: the shape of the barcode and the final filtration value in the CRS3 W group is more similar to that of the control group than the CRS1 W group. [file 12868_2016_239_MOESM4_ESM.tiff]
