# Supplementary material for: Gamma oscillation in functional brain networks is involved in the spontaneous remission of depressive behavior induced by chronic restraint stress in mice
Source: BMC Neurosci. 2016 Jan 12;17:4. doi: 10.1186/s12868-016-0239-x (PMC4710024; doi:10.1186/s12868-016-0239-x)
Supplement: Supplementary file 3 — 10.1186/s12868-016-0239-x Statistical results of the single-linkage matrices in Fig. 3a and Additional file 2: Figure S1. [file 12868_2016_239_MOESM3_ESM.pdf]

Supplementary Table 2. Statistical results of cross-correlation in Figure 3a and Supplementary Figure 1.

(Intercation between groups)

Recovery is indicated by yellow-colored box

New difference is indicated by red-colored box

Delta	1, Control; 2, CRS1W; 3, CRS3W		95% confidence interval for the true mean difference (Lower bound)	Mean difference between the groups	95% confidence interval for the true mean difference (Upper bound)	p-value	Decision (1 = Significant difference, 0 = Not Significant)
LF/RF	1	2	4.576089129	12.66666667	20.7572442	0.0007113	1
	1	3	1.348655218	8.875	16.40134478	0.0157691	1
LF/LS	1	2	-1.590577538	6.50	14.59057754	0.1435998	0
	1	3	-15.02634478	-7.50	0.026344782	0.0510471	0
LF/RS	1	2	5.980851034	14.07142857	22.16200611	0.0001354	1
	1	3	-0.82991621	6.696428571	14.22277335	0.0928703	0
LF/LP	1	2	-9.638196585	-1.54761905	6.54295849	0.8951497	0
	1	3	-18.7406305	-11.2142857	-3.687940932	0.0013915	1
LF/RP	1	2	-14.23343468	-6.14285714	1.947720395	0.1764716	0
	1	3	-16.41920192	-8.89285714	-1.366512361	0.0155111	1
LF/LV	1	2	-9.638196585	-1.54761905	6.54295849	0.8951497	0
	1	3	-18.7406305	-11.2142857	-3.687940932	0.0013915	1
LF/RV	1	2	-14.23343468	-6.14285714	1.947720395	0.1764716	0
	1	3	-16.41920192	-8.89285714	-1.366512361	0.0155111	1
RF/LS	1	2	1.123708176	9.214285714	17.30486325	0.0207678	1
	1	3	-9.937059068	-2.41071429	5.115630496	0.7332046	0
RF/RS	1	2	5.266565319	13.35714286	21.44772039	0.0003213	1
	1	3	-2.169201925	5.357142857	12.88348764	0.2174896	0
RF/LP	1	2	-9.638196585	-1.54761905	6.54295849	0.8951497	0
	1	3	-18.7406305	-11.2142857	-3.687940932	0.0013915	1
RF/RP	1	2	-14.23343468	-6.14285714	1.947720395	0.1764716	0
	1	3	-16.41920192	-8.89285714	-1.366512361	0.0155111	1
RF/LV	1	2	-9.638196585	-1.54761905	6.54295849	0.8951497	0
	1	3	-18.7406305	-11.2142857	-3.687940932	0.0013915	1
RF/RV	1	2	-14.23343468	-6.14285714	1.947720395	0.1764716	0
	1	3	-16.41920192	-8.89285714	-1.366512361	0.0155111	1
LS/RS	1	2	1.123708176	9.214285714	17.30486325	0.0207678	1
	1	3	-9.937059068	-2.41071429	5.115630496	0.7332046	0
LS/LP	1	2	-9.638196585	-1.54761905	6.54295849	0.8951497	0
	1	3	-18.7406305	-11.2142857	-3.687940932	0.0013915	1
LS/RP	1	2	-14.23343468	-6.14285714	1.947720395	0.1764716	0
	1	3	-16.41920192	-8.89285714	-1.366512361	0.0155111	1
LS/LV	1	2	-9.638196585	-1.54761905	6.54295849	0.8951497	0
	1	3	-18.7406305	-11.2142857	-3.687940932	0.0013915	1
LS/RV	1	2	-14.23343468	-6.14285714	1.947720395	0.1764716	0
	1	3	-16.41920192	-8.89285714	-1.366512361	0.0155111	1
RS/LP	1	2	-9.638196585	-1.54761905	6.54295849	0.8951497	0
	1	3	-18.7406305	-11.2142857	-3.687940932	0.0013915	1
RS/RP	1	2	-14.23343468	-6.14285714	1.947720395	0.1764716	0
	1	3	-16.41920192	-8.89285714	-1.366512361	0.0155111	1
RS/LV	1	2	-9.638196585	-1.54761905	6.54295849	0.8951497	0
	1	3	-18.7406305	-11.2142857	-3.687940932	0.0013915	1
RS/RV	1	2	-14.23343468	-6.14285714	1.947720395	0.1764716	0
	1	3	-16.41920192	-8.89285714	-1.366512361	0.0155111	1

LP/RP	1	2	-14.06676801	-5.97619048	2.114387062	0.1935359	0
	1	3	-19.16920192	-11.6428571	-4.116512361	0.0008419	1
LP/LV	1	2	4.837993891	12.92857143	21.01914897	0.0005286	1
	1	3	-2.972773353	4.553571429	12.07991621	0.3315595	0
LP/RV	1	2	-14.06676801	-5.97619048	2.114387062	0.1935359	0
	1	3	-19.16920192	-11.6428571	-4.116512361	0.0008419	1
RP/LV	1	2	-14.06676801	-5.97619048	2.114387062	0.1935359	0
	1	3	-19.16920192	-11.6428571	-4.116512361	0.0008419	1
RP/RV	1	2	-11.63819659	-3.54761905	4.54295849	0.5593416	0
	1	3	-1.490630496	6.035714286	13.56205907	0.1446075	0
LV/RV	1	2	-14.06676801	-5.97619048	2.114387062	0.1935359	0
	1	3	-19.16920192	-11.6428571	-4.116512361	0.0008419	1

**Theta**	**1, Control; 2, CRS1W; 3, CRS3W**		**95% confidence interval for the true mean difference (Lower bound)**	**Mean difference between the groups**	**95% confidence interval for the true mean difference (Upper bound)**	**p-value**	**Decision (1 = Significant difference, 0 = Not Significant)**
LF/RF	1	2	2.242755795	10.33333333	18.42391087	0.0077708	1
	1	3	3.098655218	10.625	18.15134478	0.0026974	1
LF/LS	1	2	-10.87629182	-2.78571429	5.304863252	0.6986967	0
	1	3	-19.31205907	-11.7857143	-4.259369504	0.0007093	1
LF/RS	1	2	-10.87629182	-2.78571429	5.304863252	0.6986967	0
	1	3	-19.31205907	-11.7857143	-4.259369504	0.0007093	1
LF/LP	1	2	-10.87629182	-2.78571429	5.304863252	0.6986967	0
	1	3	-19.31205907	-11.7857143	-4.259369504	0.0007093	1
LF/RP	1	2	-10.87629182	-2.78571429	5.304863252	0.6986967	0
	1	3	-19.31205907	-11.7857143	-4.259369504	0.0007093	1
LF/LV	1	2	-10.87629182	-2.78571429	5.304863252	0.6986967	0
	1	3	-19.31205907	-11.7857143	-4.259369504	0.0007093	1
LF/RV	1	2	-10.87629182	-2.78571429	5.304863252	0.6986967	0
	1	3	-19.31205907	-11.7857143	-4.259369504	0.0007093	1
RF/LS	1	2	-10.56676801	-2.47619048	5.614387062	0.7532407	0
	1	3	-19.16920192	-11.6428571	-4.116512361	0.0008419	1
RF/RS	1	2	-10.56676801	-2.47619048	5.614387062	0.7532407	0
	1	3	-19.16920192	-11.6428571	-4.116512361	0.0008419	1
RF/LP	1	2	-10.56676801	-2.47619048	5.614387062	0.7532407	0
	1	3	-19.16920192	-11.6428571	-4.116512361	0.0008419	1
RF/RP	1	2	-10.56676801	-2.47619048	5.614387062	0.7532407	0
	1	3	-19.16920192	-11.6428571	-4.116512361	0.0008419	1
RF/LV	1	2	-10.56676801	-2.47619048	5.614387062	0.7532407	0
	1	3	-19.16920192	-11.6428571	-4.116512361	0.0008419	1
RF/RV	1	2	-10.56676801	-2.47619048	5.614387062	0.7532407	0
	1	3	-19.16920192	-11.6428571	-4.116512361	0.0008419	1
LS/RS	1	2	-1.590577538	6.5	14.59057754	0.1435998	0
	1	3	-15.02634478	-7.5	0.026344782	0.0510471	0
LS/LP	1	2	-1.590577538	6.5	14.59057754	0.1435998	0
	1	3	-15.02634478	-7.5	0.026344782	0.0510471	0
LS/RP	1	2	-1.590577538	6.5	14.59057754	0.1435998	0
	1	3	-15.02634478	-7.5	0.026344782	0.0510471	0
LS/LV	1	2	-9.019148966	-0.92857143	7.162006109	0.960899	0
	1	3	-18.45491621	-10.9285714	-3.402226647	0.0019261	1
LS/RV	1	2	-9.019148966	-0.92857143	7.162006109	0.960899	0
	1	3	-18.45491621	-10.9285714	-3.402226647	0.0019261	1
RS/LP	1	2	-1.590577538	6.5	14.59057754	0.1435998	0
	1	3	-15.02634478	-7.5	0.026344782	0.0510471	0

RS/RP	1	2	-1.590577538	6.5	14.59057754	0.1435998	0
	1	3	-15.02634478	-7.5	0.026344782	0.0510471	0
RS/LV	1	2	-9.019148966	-0.92857143	7.162006109	0.960899	0
	1	3	-18.45491621	-10.9285714	-3.402226647	0.0019261	1
RS/RV	1	2	-9.019148966	-0.92857143	7.162006109	0.960899	0
	1	3	-18.45491621	-10.9285714	-3.402226647	0.0019261	1
LP/RP	1	2	-15.2572442	-7.16666667	0.923910871	0.0948189	0
	1	3	-20.52634478	-13	-5.473655218	0.0001526	1
LP/LV	1	2	-11.7572442	-3.66666667	4.423910871	0.5376487	0
	1	3	0.473655218	8	15.52634478	0.0340378	1
LP/RV	1	2	-11.7572442	-3.66666667	4.423910871	0.5376487	0
	1	3	0.473655218	8	15.52634478	0.0340378	1
RP/LV	1	2	-11.7572442	-3.66666667	4.423910871	0.5376487	0
	1	3	0.473655218	8	15.52634478	0.0340378	1
RP/RV	1	2	-11.7572442	-3.66666667	4.423910871	0.5376487	0
	1	3	0.473655218	8	15.52634478	0.0340378	1
LV/RV	1	2	-13.87629182	-5.78571429	2.304863252	0.214415	0
	1	3	-1.312059068	6.214285714	13.7406305	0.1288627	0

**Alpha**	**1, Control; 2, CRS1W; 3, CRS3W**		**95% confidence interval for the true mean difference (Lower bound)**	**Mean difference between the groups**	**95% confidence interval for the true mean difference (Upper bound)**	**p-value**	**Decision (1 = Significant difference, 0 = Not Significant)**
LF/RF	1	2	2.576089129	10.66666667	18.7572442	0.0056797	1
	1	3	2.848655218	10.375	17.90134478	0.0035356	1
LF/LS	1	2	-6.8524823	1.238095238	9.328672776	0.9315551	0
	1	3	-17.45491621	-9.92857143	-2.402226647	0.0056461	1
LF/RS	1	2	-6.8524823	1.238095238	9.328672776	0.9315551	0
	1	3	-17.45491621	-9.92857143	-2.402226647	0.0056461	1
LF/LP	1	2	-3.447720395	4.642857143	12.73343468	0.3702503	0
	1	3	-15.88348764	-8.35714286	-0.830798075	0.0250943	1
LF/RP	1	2	-3.447720395	4.642857143	12.73343468	0.3702503	0
	1	3	-15.88348764	-8.35714286	-0.830798075	0.0250943	1
LF/LV	1	2	-3.447720395	4.642857143	12.73343468	0.3702503	0
	1	3	-15.88348764	-8.35714286	-0.830798075	0.0250943	1
LF/RV	1	2	-3.447720395	4.642857143	12.73343468	0.3702503	0
	1	3	-15.88348764	-8.35714286	-0.830798075	0.0250943	1
RF/LS	1	2	-0.304863252	7.785714286	15.87629182	0.0622686	0
	1	3	-12.6156305	-5.08928571	2.437059068	0.2521835	0
RF/RS	1	2	-0.304863252	7.785714286	15.87629182	0.0622686	0
	1	3	-12.6156305	-5.08928571	2.437059068	0.2521835	0
RF/LP	1	2	-0.304863252	7.785714286	15.87629182	0.0622686	0
	1	3	-12.6156305	-5.08928571	2.437059068	0.2521835	0
RF/RP	1	2	-0.304863252	7.785714286	15.87629182	0.0622686	0
	1	3	-12.6156305	-5.08928571	2.437059068	0.2521835	0
RF/LV	1	2	-0.304863252	7.785714286	15.87629182	0.0622686	0
	1	3	-12.6156305	-5.08928571	2.437059068	0.2521835	0
RF/RV	1	2	-0.304863252	7.785714286	15.87629182	0.0622686	0
	1	3	-12.6156305	-5.08928571	2.437059068	0.2521835	0
LS/RS	1	2	-1.590577538	6.5	14.59057754	0.1435998	0
	1	3	-15.02634478	-7.5	0.026344782	0.0510471	0
LS/LP	1	2	-1.590577538	6.5	14.59057754	0.1435998	0
	1	3	-15.02634478	-7.5	0.026344782	0.0510471	0
LS/RP	1	2	-1.590577538	6.5	14.59057754	0.1435998	0
	1	3	-15.02634478	-7.5	0.026344782	0.0510471	0

LS/LV	1	2	-1.590577538	6.5	14.59057754	0.1435998	0
	1	3	-15.02634478	-7.5	0.026344782	0.0510471	0
LS/RV	1	2	-1.590577538	6.5	14.59057754	0.1435998	0
	1	3	-15.02634478	-7.5	0.026344782	0.0510471	0
RS/LP	1	2	-1.590577538	6.5	14.59057754	0.1435998	0
	1	3	-15.02634478	-7.5	0.026344782	0.0510471	0
RS/RP	1	2	-1.590577538	6.5	14.59057754	0.1435998	0
	1	3	-15.02634478	-7.5	0.026344782	0.0510471	0
RS/LV	1	2	-1.590577538	6.5	14.59057754	0.1435998	0
	1	3	-15.02634478	-7.5	0.026344782	0.0510471	0
RS/RV	1	2	-1.590577538	6.5	14.59057754	0.1435998	0
	1	3	-15.02634478	-7.5	0.026344782	0.0510471	0
LP/RP	1	2	-3.04295849	5.047619048	13.13819659	0.3092556	0
	1	3	-14.68705907	-7.16071429	0.365630496	0.0662483	0
LP/LV	1	2	-0.590577538	7.5	15.59057754	0.0759233	0
	1	3	5.223655218	12.75	20.27634478	0.0002118	1
LP/RV	1	2	-0.257244205	7.833333333	15.92391087	0.0602027	0
	1	3	4.973655218	12.5	20.02634478	0.0002923	1
RP/LV	1	2	-0.590577538	7.5	15.59057754	0.0759233	0
	1	3	5.223655218	12.75	20.27634478	0.0002118	1
RP/RV	1	2	-0.257244205	7.833333333	15.92391087	0.0602027	0
	1	3	4.973655218	12.5	20.02634478	0.0002923	1
LV/RV	1	2	-10.47152992	-2.38095238	5.709625157	0.7695009	0
	1	3	0.259369504	7.785714286	15.31205907	0.0406191	1

**Beta**	**1, Control; 2, CRS1W; 3, CRS3W**		**95% confidence interval for the true mean difference (Lower bound)**	**Mean difference between the groups**	**95% confidence interval for the true mean difference (Upper bound)**	**p-value**	**Decision (1 = Significant difference, 0 = Not Significant)**
LF/RF	1	2	3.076089129	11.16666667	19.2572442	0.0034871	1
	1	3	2.473655218	10	17.52634478	0.0052455	1
LF/LS	1	2	-2.828672776	5.261904762	13.3524823	0.2794664	0
	1	3	-15.59777335	-8.07142857	-0.54508379	0.0320574	1
LF/RS	1	2	-2.828672776	5.261904762	13.3524823	0.2794664	0
	1	3	-15.59777335	-8.07142857	-0.54508379	0.0320574	1
LF/LP	1	2	-1.590577538	6.5	14.59057754	0.1435998	0
	1	3	-15.02634478	-7.5	0.026344782	0.0510471	0
LF/RP	1	2	-1.590577538	6.5	14.59057754	0.1435998	0
	1	3	-15.02634478	-7.5	0.026344782	0.0510471	0
LF/LV	1	2	-1.590577538	6.5	14.59057754	0.1435998	0
	1	3	-15.02634478	-7.5	0.026344782	0.0510471	0
LF/RV	1	2	-1.590577538	6.5	14.59057754	0.1435998	0
	1	3	-15.02634478	-7.5	0.026344782	0.0510471	0
RF/LS	1	2	5.695136748	13.78571429	21.87629182	0.0001923	1
	1	3	-1.365630496	6.160714286	13.68705907	0.0133443	1
RF/RS	1	2	5.695136748	13.78571429	21.87629182	0.0001923	1
	1	3	-1.365630496	6.160714286	13.68705907	0.0133443	1
RF/LP	1	2	5.695136748	13.78571429	21.87629182	0.0001923	1
	1	3	-1.365630496	6.160714286	13.68705907	0.0133443	1
RF/RP	1	2	5.695136748	13.78571429	21.87629182	0.0001923	1
	1	3	-1.365630496	6.160714286	13.68705907	0.0133443	1
RF/LV	1	2	5.695136748	13.78571429	21.87629182	0.0001923	1
	1	3	-1.365630496	6.160714286	13.68705907	0.0133443	1
RF/RV	1	2	5.695136748	13.78571429	21.87629182	0.0001923	1
	1	3	-1.365630496	6.160714286	13.68705907	0.0133443	1

LS/RS	1	2	-1.590577538	6.5	14.59057754	0.1435998	0
	1	3	-15.02634478	-7.5	0.026344782	0.0510471	0
LS/LP	1	2	-1.590577538	6.5	14.59057754	0.1435998	0
	1	3	-15.02634478	-7.5	0.026344782	0.0510471	0
LS/RP	1	2	-1.590577538	6.5	14.59057754	0.1435998	0
	1	3	-15.02634478	-7.5	0.026344782	0.0510471	0
LS/LV	1	2	-1.590577538	6.5	14.59057754	0.1435998	0
	1	3	-15.02634478	-7.5	0.026344782	0.0510471	0
LS/RV	1	2	-1.590577538	6.5	14.59057754	0.1435998	0
	1	3	-15.02634478	-7.5	0.026344782	0.0510471	0
RS/LP	1	2	-1.590577538	6.5	14.59057754	0.1435998	0
	1	3	-15.02634478	-7.5	0.026344782	0.0510471	0
RS/RP	1	2	-1.590577538	6.5	14.59057754	0.1435998	0
	1	3	-15.02634478	-7.5	0.026344782	0.0510471	0
RS/LV	1	2	-1.590577538	6.5	14.59057754	0.1435998	0
	1	3	-15.02634478	-7.5	0.026344782	0.0510471	0
RS/RV	1	2	-1.590577538	6.5	14.59057754	0.1435998	0
	1	3	-15.02634478	-7.5	0.026344782	0.0510471	0
LP/RP	1	2	-0.162006109	7.928571429	16.01914897	0.0562408	0
	1	3	-12.34777335	-4.82142857	2.70491621	0.2902423	0
LP/LV	1	2	-0.423910871	7.666666667	15.7572442	0.06769	0
	1	3	5.098655218	12.625	20.15134478	0.000249	1
LP/RV	1	2	-0.876291824	7.214285714	15.30486325	0.0919108	0
	1	3	4.687940932	12.21428571	19.7406305	0.0004191	1
RP/LV	1	2	-0.423910871	7.666666667	15.7572442	0.06769	0
	1	3	5.098655218	12.625	20.15134478	0.000249	1
RP/RV	1	2	-0.876291824	7.214285714	15.30486325	0.0919108	0
	1	3	4.687940932	12.21428571	19.7406305	0.0004191	1
LV/RV	1	2	-1.804863252	6.285714286	14.37629182	0.1627265	0
	1	3	4.259369504	11.78571429	19.31205907	0.0007093	1

**Gamm**	**1, Control; 2, CRS1W; 3, CRS3W**		**95% confidence interval for the true mean difference (Lower bound)**	**Mean difference between the groups**	**95% confidence interval for the true mean difference (Upper bound)**	**p-value**	**Decision (1 = Significant difference, 0 = Not Significant)**
LF/RF	1	2	4.576089129	12.66666667	20.7572442	0.0007113	1
	1	3	1.348655218	8.875	16.40134478	0.0157691	1
LF/LS	1	2	-1.590577538	6.5	14.59057754	0.1435998	0
	1	3	-15.02634478	-7.5	0.026344782	0.0510471	0
LF/RS	1	2	-1.590577538	6.5	14.59057754	0.1435998	0
	1	3	-15.02634478	-7.5	0.026344782	0.0510471	0
LF/LP	1	2	1.552279605	9.642857143	17.73343468	0.014434	1
	1	3	-9.133487639	-1.60714286	5.919201925	0.8710856	0
LF/RP	1	2	1.552279605	9.642857143	17.73343468	0.014434	1
	1	3	-9.133487639	-1.60714286	5.919201925	0.8710856	0
LF/LV	1	2	1.552279605	9.642857143	17.73343468	0.014434	1
	1	3	-9.133487639	-1.60714286	5.919201925	0.8710856	0
LF/RV	1	2	-8.566768014	-0.47619048	7.614387062	0.9895642	0
	1	3	-10.16920192	-2.64285714	4.883487639	0.688743	0
RF/LS	1	2	5.552279605	13.64285714	21.73343468	0.0002286	1
	1	3	-1.633487639	5.892857143	13.41920192	0.1582042	0
RF/RS	1	2	5.552279605	13.64285714	21.73343468	0.0002286	1
	1	3	-1.633487639	5.892857143	13.41920192	0.1582042	0
RF/LP	1	2	2.837993891	10.92857143	19.01914897	0.0044106	1
	1	3	-6.722773353	0.803571429	8.32991621	0.9660715	0

RF/RP	1	2	2.837993891	10.92857143	19.01914897	0.0044106	1
	1	3	-6.722773353	0.803571429	8.32991621	0.9660715	0
RF/LV	1	2	2.837993891	10.92857143	19.01914897	0.0044106	1
	1	3	-6.722773353	0.803571429	8.32991621	0.9660715	0
RF/RV	1	2	-4.495339443	3.595238095	11.68581563	0.5506468	0
	1	3	-6.472773353	1.053571429	8.57991621	0.9423955	0
LS/RS	1	2	-1.590577538	6.5	14.59057754	0.1435998	0
	1	3	-15.02634478	-7.5	0.026344782	0.0510471	0
LS/LP	1	2	1.552279605	9.642857143	17.73343468	0.014434	1
	1	3	-9.133487639	-1.60714286	5.919201925	0.8710856	0
LS/RP	1	2	1.552279605	9.642857143	17.73343468	0.014434	1
	1	3	-9.133487639	-1.60714286	5.919201925	0.8710856	0
LS/LV	1	2	1.552279605	9.642857143	17.73343468	0.014434	1
	1	3	-9.133487639	-1.60714286	5.919201925	0.8710856	0
LS/RV	1	2	-8.566768014	-0.47619048	7.614387062	0.9895642	0
	1	3	-10.16920192	-2.64285714	4.883487639	0.688743	0
RS/LP	1	2	1.552279605	9.642857143	17.73343468	0.014434	1
	1	3	-9.133487639	-1.60714286	5.919201925	0.8710856	0
RS/RP	1	2	1.552279605	9.642857143	17.73343468	0.014434	1
	1	3	-9.133487639	-1.60714286	5.919201925	0.8710856	0
RS/LV	1	2	1.552279605	9.642857143	17.73343468	0.014434	1
	1	3	-9.133487639	-1.60714286	5.919201925	0.8710856	0
RS/RV	1	2	-8.566768014	-0.47619048	7.614387062	0.9895642	0
	1	3	-10.16920192	-2.64285714	4.883487639	0.688743	0
LP/RP	1	2	3.1475177	11.23809524	19.32867278	0.0032467	1
	1	3	1.29508379	8.821428571	16.34777335	0.0165659	1
LP/LV	1	2	-0.757244205	7.333333333	15.42391087	0.0849505	0
	1	3	5.348655218	12.875	20.40134478	0.00018	1
LP/RV	1	2	-10.3524823	-2.26190476	5.828672776	0.7893939	0
	1	3	0.92008379	8.446428571	15.97277335	0.0232068	1
RP/LV	1	2	3.742755795	11.83333333	19.92391087	0.0017609	1
	1	3	1.973655218	9.5	17.02634478	0.0086881	1
RP/RV	1	2	-10.68581563	-2.5952381	5.495339443	0.7325383	0
	1	3	1.17008379	8.696428571	16.22277335	0.0185648	1
LV/RV	1	2	-10.3524823	-2.26190476	5.828672776	0.7893939	0
	1	3	0.92008379	8.446428571	15.97277335	0.0232068	1
